# 1,3-Bis[(1*H*-benzotriazol-1-yl)meth­yl]-2,3-dihydro-1*H*-benzimidazole

**DOI:** 10.1107/S1600536811010701

**Published:** 2011-03-26

**Authors:** Augusto Rivera, Mauricio Maldonado, José Luis Casas, Michal Dušek, Karla Fejfarová

**Affiliations:** a Departamento de Química, Universidad Nacional de Colombia, Ciudad Universitaria, Bogotá, Colombia; b Institute of Physics ASCR, v.v.i., Na Slovance 2, 182 21 Praha 8, Czech Republic

## Abstract

In the title compound, C_21_H_18_N_8_, the two (benzotriazol-1-yl)methyl groups are located in an *anti* position with respect to the benzimidazoline moiety. The dihedral angles between the benzotriazole ring systems and the central benzimidazoline moiety are 57.03 (4) and 81.01 (3)°. The crystal packing is stabilized by two C—H⋯π inter­actions.

## Related literature

For chemical background to the synthesis of the title compound, see: Katritzky *et al.* (1990 ▶); Rivera *et al.* (2004 ▶). For related structures, see: Wang *et al.* (2008 ▶); Kuhl *et al.* (2008 ▶); Rivera *et al.* (2010 ▶). For the tautomerism of benzotriazole, see: Elguero *et al.* (2000 ▶).
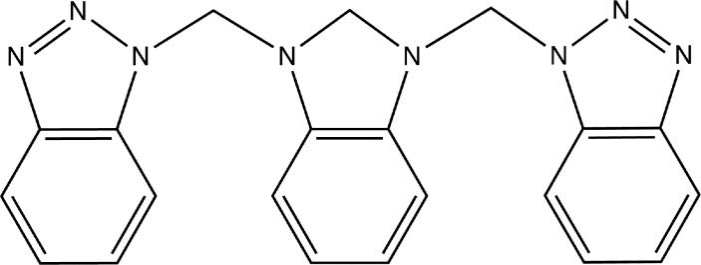


## Experimental

### 

#### Crystal data

C_21_H_18_N_8_
*M* *_r_* = 382.4
Monoclinic, 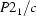

*a* = 9.0037 (2) Å
*b* = 11.5733 (3) Å
*c* = 18.0167 (4) Å
β = 103.056 (2)°
*V* = 1828.85 (8) Å^3^
*Z* = 4
Cu *K*α radiation
μ = 0.72 mm^−1^
*T* = 120 K
0.33 × 0.17 × 0.06 mm

#### Data collection

Oxford diffraction Xcalibur diffractometer with an Atlas (Gemini ultra Cu) detector
Absorption correction: multi-scan (*CrysAlis PRO*; Oxford Diffraction, 2009 ▶) *T*_min_ = 0.139, *T*_max_ = 1
27458 measured reflections
3269 independent reflections
2644 reflections with *I* > 3σ(*I*)
*R*_int_ = 0.040

#### Refinement

*R*[*F* ^2^ > 2σ(*F* ^2^)] = 0.032
*wR*(*F* ^2^) = 0.091
*S* = 1.45
3269 reflections
262 parameters
H-atom parameters constrained
Δρ_max_ = 0.13 e Å^−3^
Δρ_min_ = −0.13 e Å^−3^

### 

Data collection: *CrysAlis PRO* (Oxford Diffraction, 2009 ▶); cell refinement: *CrysAlis PRO*; data reduction: *CrysAlis PRO*; program(s) used to solve structure: *SIR2002* (Burla *et al.*, 2003 ▶); program(s) used to refine structure: *JANA2006* (Petříček *et al.*, 2007 ▶); molecular graphics: *DIAMOND* (Brandenburg & Putz, 2005 ▶); software used to prepare material for publication: *JANA2006*.

## Supplementary Material

Crystal structure: contains datablocks global, I. DOI: 10.1107/S1600536811010701/bt5499sup1.cif

Structure factors: contains datablocks I. DOI: 10.1107/S1600536811010701/bt5499Isup2.hkl

Additional supplementary materials:  crystallographic information; 3D view; checkCIF report

## Figures and Tables

**Table 1:** Hydrogen-bond geometry (Å, °) *Cg*2 is the centroid of the N3–N5/C9/C10 ring and *Cg*4 is the centroid of the C2–C7 ring.

*D*—H⋯*A*	*D*—H	H⋯*A*	*D*⋯*A*	*D*—H⋯*A*
C15—H15B⋯*Cg*2^i^	0.96	2.76	3.7131 (15)	172
C19—H19⋯*Cg*4^ii^	0.96	2.74	3.6277 (15)	154

Symmetry codes: (i) 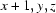; (ii) 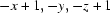.

**Fig. 1.**
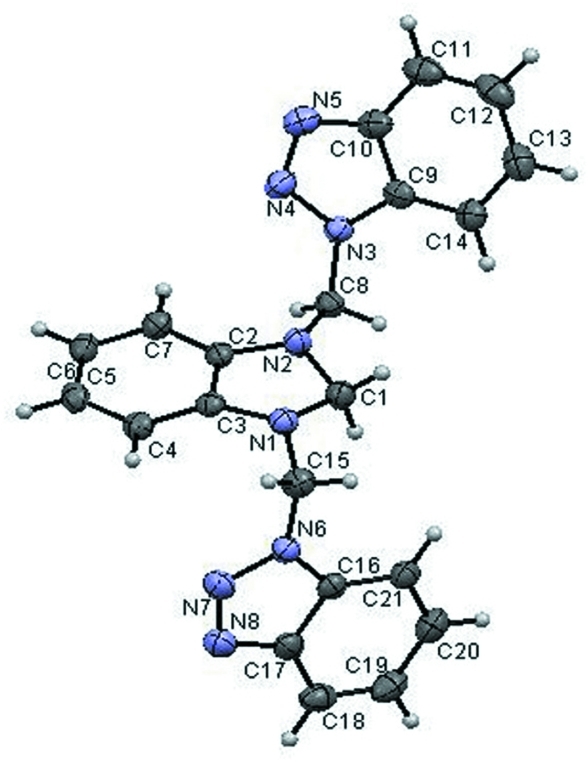
Ellipsoid plot of the title compound.

**Fig. 2.**
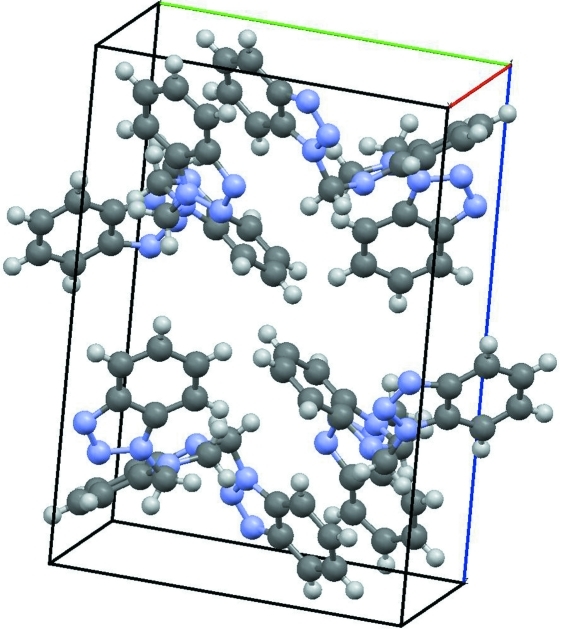
Perspective view of the crystal packing of the title compound

## supplementary crystallographic information

### Comment

Even if the synthesis of title compound I had been reported in the literature (Katritzky *et al.*, 1990), we have used an alternative route to prepare it starting from the synthetically available benzoaminal 6*H*,13*H*-5:12,7:14-dimethanedibenzo[*d,i*][1,3,6,8]tetraazecine (Rivera *et al.*, 2004) which after reaction with benzotriazole yielded a white powder which was monitored by GC—MS, ^1^H NMR, and ^13^C NMR spectra. The spectra showed the existence of the three possible isomers: 1,3-bis(benzotriazol-1-yl-methyl)-2,3-dihydrobenzimidazole (I), 1-(benzotriazol-1-yl-methyl)-2-(benzotriazol-2-yl-methyl)-2,3-dihydrobenzimidazole and 1,3-bis(benzotriazol-2-yl-methyl)-2,3-dihydrobenzimidazole as expected due to the prototropic tautomerism of benzotriazole (Elguero *et al.*, 2000). The crystal structure of before mentionated compounds has not been reported previously. So, efforts were made to crystallize all of the isomers obtained. However, only the title compound afforded single crystals suitable for structural determination and all attempts to get appropriate crystals of others isomers were unsuccessful. The crystal structure of the title compound revealed the existence of two benzotriazolyl groups in an *anti* conformation with respect to the benzimidazoline moiety. This conformation is comparable to other benzimidazoline derivatives (Wang *et al.*, 2008 and Kuhl *et al.*, 2008). Whereas the title compound was found to exist in an *anti* conformation, the skeleton of its homologous diamine possesses the *syn* conformation (Rivera *et al.*, 2010).

The X-ray data indicate the existence of an anomeric effect and confirms previous suggestions (Rivera *et al.*, 2010) as evidenced by shortened bonds lengths: N2—C1 = 1.446 (2) Å, N1—C15 = 1.431 (2) Å and N2—C8 = 1.429 (2) Å, and distorted C—N—C bond angles: C1—N1—C3= 108.3 ° and C1—N2—C2 = 109.6 °. The dihedral angles between the benzotriazolyl ring systems with the central benzimidazoline moiety are 57.03 (4)° and 81.01 (3)°. The crystal packing is stabilized by two C-H···π interactions.

### Experimental

The title compound was prepared according to the reported method (Rivera *et al.*, 2004). A suitable single-crystal (mp. 449–451 K) of the product was formed by slow evaporation of an ethyl acetate solution at room temperature.

### Refinement

All hydrogen atoms were discernible in difference Fourier maps and could be refined to reasonable geometry. According to common practice H atoms attached to C atoms were nevertheless kept in ideal positions during the refinement. The C—H distances were constrained to 0.96 Å. The isotropic atomic displacement parameters of hydrogen atoms were evaluated as 1.2**U*_eq_ of the parent atom.

### Crystal data

**Table:**

C_21_H_18_N_8_	*F*(000) = 800
*M**_r_* = 382.4	*D*_x_ = 1.389 Mg m^−^^3^
Monoclinic, *P*2_1_/*c*	Cu *K*α radiation, λ = 1.5418 Å
Hall symbol: -P 2ybc	Cell parameters from 11617 reflections
*a* = 9.0037 (2) Å	θ = 3.8–67.1°
*b* = 11.5733 (3) Å	µ = 0.72 mm^−^^1^
*c* = 18.0167 (4) Å	*T* = 120 K
β = 103.056 (2)°	Plate, colourless
*V* = 1828.85 (8) Å^3^	0.33 × 0.17 × 0.06 mm
*Z* = 4	

### Data collection

**Table:**

Oxford diffraction Xcalibur diffractometer with an Atlas (Gemini ultra Cu) detector	3269 independent reflections
Radiation source: Enhance Ultra (Cu) X-ray Source	2644 reflections with *I* > 3σ(*I*)
mirror	*R*_int_ = 0.040
Detector resolution: 10.3784 pixels mm^-1^	θ_max_ = 67.2°, θ_min_ = 4.6°
Rotation method data acquisition using ω scans	*h* = −10→10
Absorption correction: multi-scan (*CrysAlis PRO*; Oxford Diffraction, 2009)	*k* = −13→13
*T*_min_ = 0.139, *T*_max_ = 1	*l* = −21→21
27458 measured reflections	

### Refinement

**Table:**

Refinement on *F*^2^	72 constraints
*R*[*F*^2^ > 2σ(*F*^2^)] = 0.032	H-atom parameters constrained
*wR*(*F*^2^) = 0.091	Weighting scheme based on measured s.u.'s *w* = 1/[σ^2^(*I*) + 0.0016*I*^2^]
*S* = 1.45	(Δ/σ)_max_ = 0.007
3269 reflections	Δρ_max_ = 0.13 e Å^−^^3^
262 parameters	Δρ_min_ = −0.13 e Å^−^^3^
0 restraints	

### Special details

**Table:**

Experimental. CrysAlisPro, Oxford Diffraction (2009), Empirical absorption correction using spherical harmonics, implemented in SCALE3 ABSPACK scaling algorithm.
Refinement. The refinement was carried out against all reflections. The conventional *R*-factor is always based on *F*. The goodness of fit as well as the weighted *R*-factor are based on *F* and *F*^2^ for refinement carried out on *F* and *F*^2^, respectively. The threshold expression is used only for calculating *R*-factors *etc*. and it is not relevant to the choice of reflections for refinement.The program used for refinement, Jana2006, uses the weighting scheme based on the experimental expectations, see _refine_ls_weighting_details, that does not force *S* to be one. Therefore the values of *S* are usually larger than the ones from the *SHELX* program.

### Fractional atomic coordinates and isotropic or equivalent isotropic displacement parameters (Å^2^)

**Table:**

	*x*	*y*	*z*	*U*_iso_*/*U*_eq_	
N1	0.53621 (12)	0.20581 (9)	0.71929 (6)	0.0295 (4)	
N2	0.28286 (12)	0.23894 (9)	0.69788 (7)	0.0335 (4)	
N3	0.05847 (12)	0.28228 (9)	0.74331 (6)	0.0285 (3)	
N4	0.00392 (13)	0.39061 (10)	0.72735 (7)	0.0356 (4)	
N5	−0.04882 (14)	0.42890 (10)	0.78456 (7)	0.0388 (4)	
N6	0.69451 (12)	0.08507 (9)	0.65658 (6)	0.0294 (3)	
N7	0.78450 (13)	0.13645 (10)	0.61473 (7)	0.0371 (4)	
N8	0.78890 (14)	0.07188 (11)	0.55626 (7)	0.0398 (4)	
C1	0.39297 (15)	0.14738 (12)	0.72079 (8)	0.0313 (4)	
C2	0.34562 (15)	0.32489 (11)	0.66049 (7)	0.0273 (4)	
C3	0.50269 (15)	0.30412 (11)	0.67269 (7)	0.0268 (4)	
C4	0.59574 (15)	0.37697 (11)	0.64336 (7)	0.0310 (4)	
C5	0.52849 (16)	0.47226 (11)	0.60077 (8)	0.0330 (4)	
C6	0.37375 (16)	0.49275 (12)	0.58887 (8)	0.0331 (4)	
C7	0.27925 (16)	0.41919 (11)	0.61955 (8)	0.0313 (4)	
C8	0.12278 (15)	0.21954 (11)	0.68743 (8)	0.0299 (4)	
C9	0.04208 (14)	0.24901 (11)	0.81371 (7)	0.0280 (4)	
C10	−0.02855 (15)	0.34352 (12)	0.83937 (8)	0.0338 (4)	
C11	−0.07110 (17)	0.33944 (14)	0.90961 (9)	0.0422 (5)	
C12	−0.03998 (18)	0.23970 (15)	0.95043 (9)	0.0460 (6)	
C13	0.03345 (18)	0.14500 (14)	0.92440 (8)	0.0416 (5)	
C14	0.07602 (15)	0.14743 (12)	0.85558 (8)	0.0330 (4)	
C15	0.67410 (15)	0.14109 (12)	0.72642 (8)	0.0318 (4)	
C16	0.63898 (14)	−0.01708 (11)	0.62348 (7)	0.0279 (4)	
C17	0.69984 (15)	−0.02422 (12)	0.55896 (8)	0.0325 (4)	
C18	0.66863 (18)	−0.11979 (13)	0.51014 (8)	0.0402 (5)	
C19	0.57612 (18)	−0.20363 (13)	0.52823 (8)	0.0413 (5)	
C20	0.51487 (17)	−0.19509 (12)	0.59347 (8)	0.0390 (5)	
C21	0.54493 (15)	−0.10265 (11)	0.64245 (8)	0.0322 (4)	
H1a	0.37474	0.087187	0.683165	0.0376*	
H1b	0.393574	0.124273	0.772035	0.0376*	
H4	0.703349	0.362945	0.651809	0.0372*	
H5	0.590948	0.524185	0.579504	0.0396*	
H6	0.330247	0.558337	0.559148	0.0397*	
H7	0.172033	0.434108	0.61224	0.0376*	
H8a	0.103977	0.138348	0.691226	0.0358*	
H8b	0.071757	0.242837	0.636967	0.0358*	
H11	−0.119754	0.403749	0.928018	0.0507*	
H12	−0.068878	0.233577	0.998411	0.0552*	
H13	0.054267	0.077025	0.955556	0.0499*	
H14	0.125822	0.083288	0.837671	0.0396*	
H15a	0.68079	0.084238	0.765834	0.0381*	
H15b	0.759932	0.190138	0.74614	0.0381*	
H18	0.71056	−0.126041	0.465815	0.0483*	
H19	0.552197	−0.269984	0.495748	0.0496*	
H20	0.450035	−0.255859	0.604024	0.0468*	
H21	0.503676	−0.097288	0.687027	0.0386*	

### Atomic displacement parameters (Å^2^)

**Table:**

	*U*^11^	*U*^22^	*U*^33^	*U*^12^	*U*^13^	*U*^23^
N1	0.0281 (6)	0.0275 (6)	0.0335 (6)	0.0015 (4)	0.0082 (5)	0.0025 (5)
N2	0.0286 (6)	0.0297 (6)	0.0445 (7)	0.0023 (5)	0.0133 (5)	0.0086 (5)
N3	0.0274 (5)	0.0258 (6)	0.0323 (6)	−0.0002 (4)	0.0070 (5)	−0.0002 (5)
N4	0.0364 (6)	0.0281 (6)	0.0420 (7)	0.0039 (5)	0.0082 (5)	0.0003 (5)
N5	0.0383 (6)	0.0353 (7)	0.0424 (7)	0.0048 (5)	0.0085 (5)	−0.0050 (5)
N6	0.0259 (5)	0.0277 (6)	0.0349 (6)	−0.0002 (4)	0.0073 (5)	0.0008 (5)
N7	0.0297 (6)	0.0363 (6)	0.0483 (7)	−0.0006 (5)	0.0153 (5)	0.0032 (5)
N8	0.0384 (7)	0.0396 (7)	0.0457 (7)	0.0049 (5)	0.0187 (6)	0.0056 (6)
C1	0.0304 (7)	0.0295 (7)	0.0342 (7)	0.0005 (5)	0.0074 (6)	0.0027 (6)
C2	0.0310 (7)	0.0255 (6)	0.0260 (6)	−0.0034 (5)	0.0077 (5)	−0.0031 (5)
C3	0.0307 (7)	0.0249 (6)	0.0246 (6)	−0.0009 (5)	0.0060 (5)	−0.0045 (5)
C4	0.0297 (7)	0.0303 (7)	0.0329 (7)	−0.0054 (6)	0.0069 (6)	−0.0047 (6)
C5	0.0395 (8)	0.0288 (7)	0.0312 (7)	−0.0093 (6)	0.0091 (6)	−0.0025 (5)
C6	0.0429 (8)	0.0252 (7)	0.0303 (7)	−0.0023 (6)	0.0063 (6)	0.0007 (5)
C7	0.0314 (7)	0.0291 (7)	0.0325 (7)	0.0002 (6)	0.0050 (6)	−0.0009 (5)
C8	0.0297 (7)	0.0293 (7)	0.0319 (7)	−0.0022 (5)	0.0098 (6)	−0.0021 (5)
C9	0.0221 (6)	0.0322 (7)	0.0289 (7)	−0.0054 (5)	0.0040 (5)	−0.0043 (5)
C10	0.0281 (7)	0.0354 (8)	0.0373 (8)	−0.0036 (6)	0.0060 (6)	−0.0084 (6)
C11	0.0368 (8)	0.0518 (10)	0.0393 (8)	−0.0049 (7)	0.0110 (6)	−0.0134 (7)
C12	0.0442 (9)	0.0632 (11)	0.0324 (8)	−0.0140 (8)	0.0127 (7)	−0.0093 (7)
C13	0.0446 (8)	0.0452 (9)	0.0328 (7)	−0.0131 (7)	0.0043 (6)	0.0017 (6)
C14	0.0315 (7)	0.0322 (7)	0.0337 (7)	−0.0052 (6)	0.0037 (6)	−0.0016 (6)
C15	0.0285 (7)	0.0337 (7)	0.0309 (7)	0.0008 (5)	0.0020 (5)	−0.0018 (6)
C16	0.0256 (6)	0.0279 (7)	0.0286 (6)	0.0046 (5)	0.0023 (5)	0.0026 (5)
C17	0.0316 (7)	0.0328 (7)	0.0334 (7)	0.0082 (6)	0.0081 (6)	0.0056 (6)
C18	0.0484 (9)	0.0413 (8)	0.0305 (7)	0.0142 (7)	0.0079 (6)	0.0009 (6)
C19	0.0519 (9)	0.0317 (8)	0.0351 (8)	0.0080 (7)	−0.0010 (7)	−0.0050 (6)
C20	0.0423 (8)	0.0276 (7)	0.0434 (9)	0.0004 (6)	0.0021 (7)	0.0018 (6)
C21	0.0345 (7)	0.0282 (7)	0.0334 (7)	0.0022 (6)	0.0067 (6)	0.0030 (6)

### Geometric parameters (Å, °)

**Table:**

N1—C1	1.4619 (18)	C6—H6	0.96
N1—C3	1.4057 (16)	C7—H7	0.96
N1—C15	1.4307 (17)	C8—H8a	0.96
N2—C1	1.4461 (17)	C8—H8b	0.96
N2—C2	1.3908 (18)	C9—C10	1.395 (2)
N2—C8	1.4286 (17)	C9—C14	1.3931 (18)
N3—N4	1.3535 (15)	C10—C11	1.403 (2)
N3—C8	1.4626 (19)	C11—C12	1.363 (2)
N3—C9	1.3651 (18)	C11—H11	0.96
N4—N5	1.3058 (19)	C12—C13	1.414 (2)
N5—C10	1.3797 (19)	C12—H12	0.96
N6—N7	1.3618 (18)	C13—C14	1.378 (2)
N6—C15	1.4635 (18)	C13—H13	0.96
N6—C16	1.3668 (16)	C14—H14	0.96
N7—N8	1.2994 (18)	C15—H15a	0.96
N8—C17	1.3784 (19)	C15—H15b	0.96
C1—H1a	0.96	C16—C17	1.395 (2)
C1—H1b	0.96	C16—C21	1.3948 (19)
C2—C3	1.4020 (18)	C17—C18	1.402 (2)
C2—C7	1.3759 (17)	C18—C19	1.365 (2)
C3—C4	1.376 (2)	C18—H18	0.96
C4—C5	1.4006 (18)	C19—C20	1.410 (2)
C4—H4	0.96	C19—H19	0.96
C5—C6	1.381 (2)	C20—C21	1.3743 (19)
C5—H5	0.96	C20—H20	0.96
C6—C7	1.402 (2)	C21—H21	0.96
			
C1—N1—C3	108.31 (10)	N3—C8—H8b	109.4707
C1—N1—C15	120.50 (11)	H8a—C8—H8b	106.7384
C3—N1—C15	122.84 (12)	N3—C9—C10	103.88 (11)
C1—N2—C2	109.58 (11)	N3—C9—C14	133.21 (13)
C1—N2—C8	121.91 (11)	C10—C9—C14	122.87 (13)
C2—N2—C8	123.83 (10)	N5—C10—C9	108.70 (13)
N4—N3—C8	119.65 (11)	N5—C10—C11	130.59 (14)
N4—N3—C9	110.39 (11)	C9—C10—C11	120.67 (13)
C8—N3—C9	129.96 (11)	C10—C11—C12	116.76 (15)
N3—N4—N5	109.02 (11)	C10—C11—H11	121.6181
N4—N5—C10	108.00 (12)	C12—C11—H11	121.6189
N7—N6—C15	119.24 (10)	C11—C12—C13	122.14 (16)
N7—N6—C16	109.93 (11)	C11—C12—H12	118.9315
C15—N6—C16	130.82 (12)	C13—C12—H12	118.9322
N6—N7—N8	109.19 (11)	C12—C13—C14	121.91 (14)
N7—N8—C17	108.11 (13)	C12—C13—H13	119.0442
N1—C1—N2	101.92 (10)	C14—C13—H13	119.044
N1—C1—H1a	109.4711	C9—C14—C13	115.65 (13)
N1—C1—H1b	109.4711	C9—C14—H14	122.176
N2—C1—H1a	109.4717	C13—C14—H14	122.1781
N2—C1—H1b	109.4706	N1—C15—N6	115.45 (10)
H1a—C1—H1b	116.0962	N1—C15—H15a	109.4716
N2—C2—C3	107.89 (10)	N1—C15—H15b	109.4715
N2—C2—C7	130.71 (12)	N6—C15—H15a	109.4709
C3—C2—C7	121.37 (13)	N6—C15—H15b	109.4707
N1—C3—C2	107.78 (11)	H15a—C15—H15b	102.7515
N1—C3—C4	131.17 (12)	N6—C16—C17	103.94 (12)
C2—C3—C4	121.00 (11)	N6—C16—C21	133.61 (13)
C3—C4—C5	117.88 (13)	C17—C16—C21	122.45 (12)
C3—C4—H4	121.0592	N8—C17—C16	108.83 (12)
C5—C4—H4	121.0603	N8—C17—C18	130.57 (14)
C4—C5—C6	121.08 (13)	C16—C17—C18	120.59 (13)
C4—C5—H5	119.4594	C17—C18—C19	117.23 (15)
C6—C5—H5	119.4597	C17—C18—H18	121.3823
C5—C6—C7	121.00 (12)	C19—C18—H18	121.3839
C5—C6—H6	119.499	C18—C19—C20	121.59 (14)
C7—C6—H6	119.4995	C18—C19—H19	119.2076
C2—C7—C6	117.65 (13)	C20—C19—H19	119.2074
C2—C7—H7	121.1743	C19—C20—C21	122.17 (14)
C6—C7—H7	121.1739	C19—C20—H20	118.9165
N2—C8—N3	112.07 (10)	C21—C20—H20	118.9176
N2—C8—H8a	109.4717	C16—C21—C20	115.97 (14)
N2—C8—H8b	109.4715	C16—C21—H21	122.0168
N3—C8—H8a	109.4707	C20—C21—H21	122.0158
			
C3—N1—C1—N2	−20.63 (13)	N7—N8—C17—C16	−0.40 (16)
C15—N1—C1—N2	−169.70 (11)	N7—N8—C17—C18	−179.17 (15)
C1—N1—C3—C2	14.16 (14)	N2—C2—C3—N1	−1.19 (14)
C1—N1—C3—C4	−168.50 (13)	N2—C2—C3—C4	−178.85 (12)
C15—N1—C3—C2	162.35 (11)	C7—C2—C3—N1	176.99 (12)
C15—N1—C3—C4	−20.3 (2)	C7—C2—C3—C4	−0.67 (19)
C1—N1—C15—N6	80.80 (15)	N2—C2—C7—C6	179.09 (13)
C3—N1—C15—N6	−63.69 (16)	C3—C2—C7—C6	1.39 (19)
C2—N2—C1—N1	20.13 (14)	N1—C3—C4—C5	−177.23 (13)
C8—N2—C1—N1	176.70 (12)	C2—C3—C4—C5	−0.19 (19)
C1—N2—C2—C3	−12.47 (14)	C3—C4—C5—C6	0.28 (19)
C1—N2—C2—C7	169.58 (14)	C4—C5—C6—C7	0.5 (2)
C8—N2—C2—C3	−168.51 (12)	C5—C6—C7—C2	−1.3 (2)
C8—N2—C2—C7	13.6 (2)	N3—C9—C10—N5	−0.99 (15)
C1—N2—C8—N3	114.09 (13)	N3—C9—C10—C11	176.99 (13)
C2—N2—C8—N3	−92.71 (15)	C14—C9—C10—N5	−178.84 (13)
C8—N3—N4—N5	179.34 (11)	C14—C9—C10—C11	−0.9 (2)
C9—N3—N4—N5	−0.58 (15)	N3—C9—C14—C13	−176.33 (14)
N4—N3—C8—N2	90.24 (14)	C10—C9—C14—C13	0.8 (2)
C9—N3—C8—N2	−89.85 (16)	N5—C10—C11—C12	177.47 (15)
N4—N3—C9—C10	0.95 (14)	C9—C10—C11—C12	0.0 (2)
N4—N3—C9—C14	178.48 (14)	C10—C11—C12—C13	0.9 (2)
C8—N3—C9—C10	−178.96 (13)	C11—C12—C13—C14	−0.9 (3)
C8—N3—C9—C14	−1.4 (2)	C12—C13—C14—C9	0.1 (2)
N3—N4—N5—C10	−0.08 (16)	N6—C16—C17—N8	0.44 (15)
N4—N5—C10—C9	0.69 (16)	N6—C16—C17—C18	179.36 (13)
N4—N5—C10—C11	−177.02 (15)	C21—C16—C17—N8	−179.23 (12)
C15—N6—N7—N8	178.98 (11)	C21—C16—C17—C18	−0.3 (2)
C16—N6—N7—N8	0.10 (15)	N6—C16—C21—C20	−179.76 (14)
N7—N6—C15—N1	99.09 (14)	C17—C16—C21—C20	−0.2 (2)
C16—N6—C15—N1	−82.29 (17)	N8—C17—C18—C19	179.21 (15)
N7—N6—C16—C17	−0.33 (14)	C16—C17—C18—C19	0.6 (2)
N7—N6—C16—C21	179.29 (14)	C17—C18—C19—C20	−0.3 (2)
C15—N6—C16—C17	−179.05 (13)	C18—C19—C20—C21	−0.2 (2)
C15—N6—C16—C21	0.6 (2)	C19—C20—C21—C16	0.5 (2)
N6—N7—N8—C17	0.19 (15)		

### Hydrogen-bond geometry (Å, °)

**Table:**

Cg2 is the centroid of the N3–N5/C9/C10 ring and Cg4 is the centroid of the C2–C7 ring.

**Table:**

*D*—H···*A*	*D*—H	H···*A*	*D*···*A*	*D*—H···*A*
C15—H15B···Cg2^i^	0.96	2.76	3.7131 (15)	172
C19—H19···Cg4^ii^	0.96	2.74	3.6277 (15)	154

Symmetry codes: (i) *x*+1, *y*, *z*; (ii) −*x*+1, −*y*, −*z*+1.
